# Health-Related Quality of Life in Pregnant Women during the First Trimester in Northern Spain: A Descriptive Cross-Sectional Study

**DOI:** 10.3390/healthcare11101424

**Published:** 2023-05-14

**Authors:** Cristian Martín-Vázquez, Rubén García-Fernández, Natalia Calvo-Ayuso, María Cristina Martínez-Fernández, Cristina Liébana-Presa, José David Urchaga-Litago

**Affiliations:** 1 Department of Nursing and Physiotherapy, Campus de Ponferrada, Universidad de León, 24401 León, Spain; cmartv@unileon.es (C.M.-V.); ncala@unileon.es (N.C.-A.); 2 SALBIS Research Group, Faculty of Health Sciences, Department of Nursing and Physiotherapy, Campus de Ponferrada, Universidad de León, 24401 León, Spain; mmartf@unileon.es (M.C.M.-F.); cliep@unileon.es (C.L.-P.); 3 Faculty of Communication, Pontifical University of Salamanca, 37002 Salamanca, Spain; jdurchagali@upsa.es

**Keywords:** pregnancy, SF-36, quality of life, health

## Abstract

Background: Achieving the optimal quality of life is currently a health challenge for the world’s population. Pregnancy is a stressful period of life that affects women’s quality of life. Aims: This study aimed to describe and analyse the health-related quality of life in pregnant women during their first trimester in a health area in the north of Spain. Methods: A cross-sectional descriptive study was carried out. A total of 359 women completed the 36-Item Short-Form Health Survey. Results: The sample consisted of 57.9% primiparous women, 30% had experienced a previous abortion, and 7.2% were foreign women. The mean age was 33.53 years. The sum of the physical and mental component values was below 50 points. Notably, 4.17% of women reported a worsening of their health in the last year, and 28.69% had an increased depression risk. Conclusion: Being a foreigner, prenatal abortion, previous caesarean section, previous children, or assisted reproduction techniques are the variables that have a negative association with some dimensions of quality of life in pregnant women.

## 1. Introduction

Quality of life (QoL) has become a major issue in our society. The World Health Organisation (WHO) has included it in the Sustainable Development Goals (SDGs), specifically in the third goal “health and well-being” [1]. In 1993, the WHO defined QoL as “an individual’s perception of his or her position in life within the cultural context and value system in which he or she lives and for his or her goals, expectations, norms and concerns”.

This definition reflects the quality of life as perceived satisfaction in different areas of life. It is an individual and subjective concept in which each person has his or her perception of quality of life according to his or her desires, wishes, satisfactions, and life goals [2]. More specifically, the quality of life in the perinatal stage is defined as “a multidimensional concept that refers to a woman’s perception of the influence of pregnancy, childbirth, and postpartum on her physical, mental, emotional, and social functioning” [3].

Quality of life has been classically defined by two essential components, one physical and one mental [4,5]. It is influenced by factors inherent to the human being and external agents that include one’s physical health, mental health, level of dependency, social relations, and relationship with the environment. Therefore, the approach must be multidimensional and not isolated [2].

Pregnancy can cause clinical manifestations, such as vomiting and tiredness, which may negatively impact women’s quality of life, particularly in the first trimester [6]. Factors such as drug use, late pregnancy, low socioeconomic status, and poor obstetric history also contribute to a reduced perception of quality of life [7].

In Spain, according to a report by the National Institute of Statistics, QoL decreased in 2020 after improving continuously since 2014. The multidimensional indicator of quality of life (MQoL) stood at 101.71 points in 2020, compared with 102.06 in 2019. Major decreases were observed in health, environment, surroundings, and material living conditions [8].

According to Eurostat data, in 2020, 73% of the Spanish population considered their health to be good or very good, an increase of more than two points from 2019 (75.3%). In 2021, the data reflect a further drop of almost two points and a decreasing trend in this item (71.2%). Despite this, Spain ranks 13th out of the 27 Eurozone countries (2000), behind Nordic countries such as Norway and Finland; Central European countries such as Belgium, Switzerland, and Austria; and Mediterranean countries such as Italy, Greece, Malta, and Cyprus [9].

COVID-19 significantly impacted the quality of life, with pregnant women being more vulnerable to infections, particularly SARS-CoV-2, and experiencing increased mental and psychological health issues. The pandemic heightened concerns about maternal and child health and led to reduced social performance, vitality, and quality of life for pregnant women [10,11,12,13,14].

The effectiveness of nursing interventions to improve women’s quality of life during pregnancy has been documented in international research [15,16]. However, there are few recent studies on the quality of life during pregnancy performed in Spain [17,18].

Due to the above factors, the present study is proposed with the general objective of describing and analysing the health-related quality of life in pregnant women during the first trimester in an area in the north of Spain. Secondly, we sought to study the variation in the quality of life according to sociodemographic and gynaecological–obstetric factors.

## 2. Materials and Methods

### 2.1. Study Design and Procedure 

A descriptive cross-sectional study was conducted. Participants were enrolled during the first obstetric consultation, between the sixth and eighth week of gestation, in a hospital in a health area in the north of Spain. At this appointment, they were informed of the study. Those who wished to participate were given the informed consent document and a sheet with instructions for completing the questionnaire around the tenth week of gestation. Data collection was carried out between 15 September 2021 and 14 September 2022.

### 2.2. Sample

**Figure 1:**
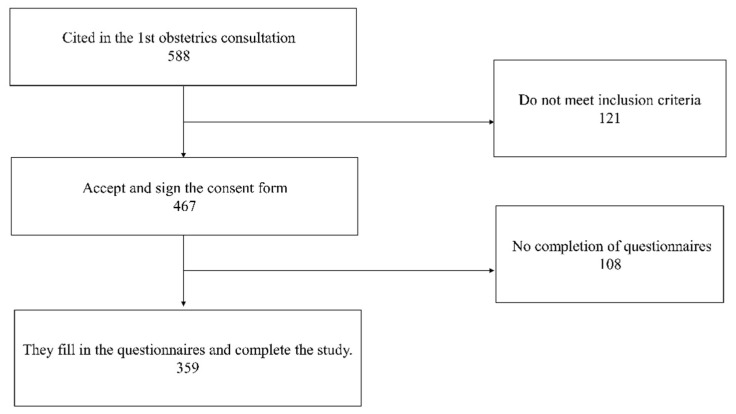
Flowchart of the study population.

The total sample consisted of 359 women (Figure 1). Inclusion criteria were set as pregnant women of legal age. Women with a personal history of anxiety, depression, or psychiatric illness; language barrier; difficulty in completing the questionnaire either due to lack of knowledge or lack of technological means; and lack of consent or refusal to participate in the study were excluded. Finally, 461 participants met the inclusion criteria. Out of these 461 participants, 108 did not complete the questionnaires, and therefore their participation in the study was rejected. During the year 2021, a total of 501 women gave birth in the hospital under study. For a confidence level of 95% and the worst-case scenario (p = Q), the sampling error was 1.2%.

All the subjects voluntarily signed the informed consent form, which was prepared following the Declaration of Helsinki (World Medical Association, 2013) and the European Union’s Good Clinical Practice Directive (Directive 2005/28/EC). The research protocol was approved by the Ethics Committee of the León and Bierzo Health Areas (reference code 21124) and by the ethics committee of the University of León (ETICA-ULE-033-2021).

### 2.3. Instrument

Data were collected using a questionnaire that included sociodemographic data (age, marital status, nationality, and area where she lives) and obstetric–gynaecological data (date of last menstrual period, gestational formula, obstetric history, type of breastfeeding desired, and pregnancy outcome of spontaneous or artificial conception).

Health-related quality of life was assessed using the 36-Item Short-Form Health Survey (SF-36 v2), developed in 1993 by Are and validated for the Spanish population by Alonso et al. in 1995. It is an instrument designed to evaluate the state of health perceived by the individual and includes 36 items that analyse 8 dimensions (physical functioning, role physical, bodily pain, general health, vitality, social functioning, role emotional, and mental health). In addition, this questionnaire has a health transition item (not used for the calculation of any of the dimensions) that provides information on how the individual’s health has been in the last year. Besides the eight dimensions above, two main health components can be extracted: the physical component summary (PCS) and the mental component summary (MCS). Scores for these dimensions range from 0 to 100. Values above 50 points indicate a better health condition than the average of the reference population. These scores were calculated following the recommendations of Vilagut et al. [19]. Notably, 96% of the scales analysed by Vilagut et al. in 2005 exceeded the proposed standard of reliability (Cronbach’s α) of 0.7 [2,3,4,5].

### 2.4. Statistical Analysis 

The data were analysed using SPSS Statistics v28.0 software (IBM, Armonk, NY, USA). A descriptive analysis of the sample participants was performed (frequencies and percentages), and the level of perception of QoL was analysed in all its dimensions and the physical and mental sums. The Kolmogorov–Smirnov test with Lilliefors correction was used to test the distributions of the numerical variables, revealing the absence of normality in all the variables under study. Subsequently, the differences between QoL and the study variables were analysed using the non-parametric Mann–Whitney U test. In the bivariate analysis, Spearman’s correlation was used to examine the relationship between maternal age and QoL. The level of significance in this study was set at *p* < 0.05.

## 3. Results

### 3.1. Sample

Table 1 provides a comprehensive overview of the sociodemographic and obstetric–gynaecological characteristics of the study sample. The findings indicate that a significant proportion of the participants were primiparous, accounting for 57.9% of the total sample. In contrast, 42.1% of the women had previously given birth to one or more children. Regarding marital status, the majority of the participants, 81.1%, reported being married or cohabiting, while 18.9% were either single or widowed.

In terms of obstetric history, 69.9% did not have a history of previous miscarriage, while 108 women had experienced a miscarriage in previous pregnancies. Another important obstetric history is the presence of a previous caesarean section; in this regard, we observed that 13.38% of the sample had undergone a caesarean section in previous pregnancies.

Regarding the mode of conception, the majority of the participants, 92.8%, reported achieving the current pregnancy through spontaneous conception, indicating that they were able to conceive naturally without the assistance of reproductive technologies. However, it is worth noting that a small proportion, 7.2%, sought specialised medical interventions such as artificial insemination or in vitro fertilisation to conceive, reflecting the diverse paths to pregnancy experienced by the participants.

Additionally, regarding the decision on infant feeding, 48.2% of the sample had made the choice to exclusively breastfeed their future baby, while 21.4% had not yet made a decision.

Geographically, the sample composition was diverse, with 29.5% of the participants residing in rural areas and 70.5% living in urban settings. This distribution reflects the demographic representation of both rural and urban populations within the study region. Additionally, it is noteworthy that the majority of the participants, 92.8%, were of Spanish nationality.

### 3.2. Internal Consistency

The statistical analysis reflects a broad internal consistency of the SF-36 questionnaire, with a Cronbach’s alpha value of 0.90 and a McDonald’s omega of 0.97. For all dimensions except for social function, the internal consistency was good, with Cronbach’s alpha values between 0.71 and 0.98. The results of this analysis are shown in Table 2.

### 3.3. Quality of Life

Table 3 presents the average and standard deviation of the quality of life dimensions assessed by the SF-36 questionnaire, as well as the physical and mental summary components. The dimensions with the lowest scores were vitality, bodily pain, and role physical, with mean values of 44.05, 48.62, and 51.95, respectively. On the other hand, the dimension with the highest score by far was physical function, with a mean score of 83.97. Analysing the physical and mental summary components, we observe that both had mean scores below the 50th percentile; specifically, these values were 46.32 for the physical component summary (PCS) and 45.21 for the mental component summary (MCS), indicating lower scores in the mental aspect than in the physical aspect.

Considering item 2 of the SF-36, which compares the current health of the women with that of the previous year, 61.6% of the women reported having improved their health to the previous year, whereas 4.17% reported a worsening of their health in the last year.

### 3.4. Maternal Age and Quality of Life

Another variable analysed was maternal age in the first trimester. We observed a mean age of 33.53 years with a standard deviation of 4.81. The age range of the sample was between 20 and 48 years. In terms of the older age group, the percentage of women over 39 years of age corresponded to 8.9% of the sample.

The Spearman correlation analysis revealed positive correlations in all the variables and summary components, except for the physical function dimension. As seen in Table 4, these positive correlations were statistically significant in the bodily pain and general health dimensions, with the *p*-value in the latter being less than 0.01.

We did not observe any cutoff point at which the difference in means of quality of life dimensions between older women and younger women was statistically significant.

### 3.5. Quality of Life and Qualitative Variables

Table 5 presents the mean differences between groups on the physical and mental dimensions and summary components of the SF-36 questionnaire.

Primiparous women obtained higher scores than multiparous women in all dimensions, as well as in the physical component summary (PCS) and mental component summary (MCS). This difference in means was statistically significant in vitality, social functioning, and emotional role.

Analysing women with a history of previous caesarean section, we observed that women without this prior surgery had higher scores in all dimensions and the physical component summary than women with a previous caesarean section, except for bodily pain and MCS. This difference in means was statistically significant in the physical-role dimension.

Women who did not experience a previous pregnancy loss (miscarriage) obtained higher scores than women with a history of miscarriage in all dimensions and PCS. This difference was statistically significant (*p* < 0.01) in the social functioning, emotional role, and mental health dimensions.

In terms of nationality, Spanish women obtained higher scores in all dimensions and MCS compared with foreign women. This difference was statistically significant in the social functioning, emotional role, and mental health dimensions.

When examining the mode of conception, women who achieved spontaneous conception without assisted reproductive techniques had higher scores in the physical functioning dimension. This difference was statistically significant (*p* < 0.01).

No significant differences were found between the quality of life and marital status, breastfeeding intention, and area of residence.

## 4. Discussion

This study analyses the current situation in a health area in the north of Spain for the self-perceived quality of life of pregnant women in their first trimester, as measured using the SF-36 health questionnaire. The participants in this study had scores above the 50th percentile in physical function, physical role, social function, emotional role, and mental health. In contrast, scores below the 50th percentile were obtained in bodily pain and vitality. In addition, the physical and mental summary components scored 45.32 and 46.32 points, respectively.

These findings contrast with those obtained in Granada (Spain) in 2016, where it was observed that except for emotional role, the mean scores of its dimensions and sums were higher, with a mean difference of more than 20 points in the social function, bodily pain, and vitality dimensions [6].

Given that this population is like the present one, it seems that the quality of life among Spanish women in the first trimester has worsened in recent years. On the other hand, the data from our sample show a difference of 18 points higher in the emotional-role dimension. This indicates that the women in the present study have fewer problems with work and other daily activities due to emotional problems [4].

Another multicentre study in Spain in 2019 examined the quality of life during the third trimester of pregnancy. The data from this study reflect a score of 48.9 in the mental summary and 36.2 in the physical summary; these data can be explained because, in the third trimester, the physical symptoms related to maternal weight worsen, which explains the poor quality of life in the physical component. On the other hand, and in comparison with the present study, a worsening of the mental health component in Spanish pregnant women was observed [7].

Chang et al. analysed the quality of life and its relationship with obstetric factors and found that women who underwent assisted reproductive techniques scored lower on the physical-function dimension. This is probably because they experienced difficulties with conception; however, the mental health components of quality of life were not modified in either of the groups [8]. These results are in line with the present study in which lower scores were observed in the physical-function dimension in women who obtained a pregnancy with assisted reproductive techniques compared with women who had a spontaneous pregnancy.

Furthermore, in their studies, Gameiro et al. (2010) revealed that women with spontaneous pregnancies obtain better quality-of-life scores in terms of mental health [9]. Another Italian study compared the quality of life using the SF-36 health questionnaire between women with spontaneous pregnancies and women with assisted reproductive techniques. In line with the present investigation, these authors concluded that women with spontaneous pregnancies have a better quality of life in the dimensions of physical function, physical role, vitality, and social function [10].

Alzboon et al. (2019) described the factors influencing pregnant women’s quality of life in northern Jordan. In this study, women with low parity had better quality-of-life scores than women with high parity [11]. Similar findings were found in another study in Lithuania [12]. The present investigation confirms this hypothesis, since, in all the dimensions analysed in the SF-36, we observed higher scores in primiparous women than in multiparous women. In contrast, Chang et al. (2014) found that multiparous women have a better quality of life than primiparous women [8].

According to Rodriguez-Blanque et al. (2020), values below 42 points on the mental summary component (MCS) indicate an increased risk of depression [6]. A study in China published in 2021 that analysed the quality of life during gestation concluded that the risk of depression remained constant during all trimesters but always exceeded 25% of the population [13]. These data are consistent with the results of the present study. We found that 28.69% of the pregnant women in the sample were at risk of depression in the first trimester of gestation.

The data collected in the present study showed no statistically significant difference in means between older women and quality of life. A positive and statistically significant correlation was observed between age and general health and bodily pain. These findings contrast with the study of Liu et al. (2020), in which they found lower quality of life in women over 30 years of age than in younger age groups [14].

An additional possible reason for the low quality of life scores of the study sample compared with previous years may be the presence of COVID-19 fear. Numerous studies have shown that COVID-19 behaves as a source of fear, stress, and anxiety, and as a major factor affecting people’s health, well-being, and quality of life [15,16,17]. An Iranian study in 2020 analysed the quality of life and other mental health variables, such as stress, anxiety, and depression. The scores recorded in their sum of the physical and mental quality of life were 22 points higher than those found in our population. This confirms that fear of COVID-19 causes a decline in the quality of life in pregnant women [18].

Another recent study showed the importance of partners as a significant predictor of stress, anxiety, and quality of life [16]. Nevertheless, this opens up an interesting area of study, namely the active participation of partners in pregnancy not only as a protective factor against mental illness but also as a significant factor in improving the quality of life for women during pregnancy.

The main limitation of this study was that it was a cross-sectional study in a single health area in the north of Spain. Moreover, the present study was undertaken only in the first trimester of gestation, so the results could not be extrapolated to women in any other trimester. Our results cannot be generalised to women with a history of mental pathology, since none of the respondents had a history of mental illness.

This opens up new lines of research that we want to develop:

1. We plan to perform a longitudinal analysis throughout pregnancy and postpartum and analyse how the quality of life evolves throughout the perinatal stage;
2. We plan to carry out a multicentre study with the aim of including a representative sample of the entire Spanish population in order to draw conclusions at an international level;
3. We plan to analyse the quality of life with other variables of interest such as social support, resilience, stress, or anxiety.

## 5. Conclusions

The present study shows how the health-related quality of life in pregnant women in their first trimester in a northern health centre in Spain is modified in some dimensions based on gynaecological–obstetric and sociodemographic variables. Multiparous women had lower scores in the vitality dimension. Social-role scores were lower in multiparous women and women with previous miscarriages history. Emotional-role scores were higher in primiparous women, women without previous miscarriages, and Spanish pregnant women. Worse scores were observed in terms of physical role in women with a history of a caesarean section. Mental health was worse in women with prior abortions and in the foreign population. On the other hand, we found that 28.69% of the sample presented a risk of depression during the first trimester.

## Figures and Tables

**Table 1:** Descriptive analysis.

Sociodemographic and Gynecobstetric Variables		N = 359 (100%)
Parity	Primipara	208 (57.9%)
	Multipara	151 (42.1%)
Marital status	Married/cohabiting	291 (81.1%)
	Single/widowed	68 (18.9%)
Previous abortions	None	251(69.9%)
	One or more	108 (30.1%)
Breastfeeding intention	I have not thought about it yet	77 (21.4%)
	Mixed breastfeeding	88 (24.5%)
	Breastfeeding	173 (48.2%)
	Artificial breastfeeding	21 (5.8%)
Area of residence	Rural	106 (29.5%)
	Urban	253 (70.5%)
Pregnancy	Spontaneous	333 (92.8%)
	Assisted reproduction	26 (7.2%)
Nationality	Spanish	333 (92.8%)
	Foreign	26 (7.2%)
Previous caesarean section	Yes	48 (13.38%)
	No	311 (86.62)

**Table 2:** Internal consistency analysis.

Dimension	Alfa Cronbach	Omega McDonald
Physical functioning	0.89	0.90
Role physical	0.90	0.90
Bodily pain	0.98	*
General Health	0.71	0.73
Vitality	0.84	0.82
Social functioning	0.58	*
Role emotional	0.90	0.90
Mental health	0.84	0.85
SF-36	0.90	0.97

* Not assessable because it is made up of two factors.

**Table 3:** Descriptive statistics of 36-Item Short-Form Health Survey.

Dimension	Mean	SD	Median (R)
Physical functioning	83.97	19.29	90 (0–100)
Role physical	51.95	43.66	50 (0–100)
Bodily pain	48.62	28.18	52 (12–100)
General Health	77.80	17.53	80 (22–100)
Vitality	44.05	18.65	40 (10–90)
Social functioning	63.54	26.57	62.5 (0–100)
Role emotional	65.55	43.21	100 (0–100)
Mental health	65.16	18.74	68 (20–92)
PCS	46.32	8.44	47 (19–70)
MCS	45.32	7.31	46 (23–66)

SD: standard deviation; R: range; PCS: physical component summary; MCS: mental component summary.

**Table 4:** Pearson correlations between age and 36-Item Short-Form Health Survey.

Dimension	Coefficient	*p*-Value
Physical functioning	−0.01	0.85
Role—physical	0.06	0.22
Bodily pain	0.14	0.01 *
General health	0.15	<0.01 *
Vitality	0.06	0.23
Social functioning	0.01	0.79
Role—emotional	0.09	0.09
Mental health	0.03	0.54
PCS	0.07	0.17
MCS	0.04	0.40

* p-value < 0.05.

**Table 5:** Mann–Whitney U test between the quality of life and qualitative variables.

	PF	RP	BP	GH	VT	SF	RE	MH	PCS	MCS
	M (SD)	M (SD)	M (SD)	M (SD)	M (SD)	M (SD)	M (SD)	M (SD)	M (SD)	M (SD)
**Primipara**	84.30 (18.65)	55.41 (42.04)	49.03 (27.80)	79.21 (16.35)	45.94 * (18.46)	65.80 * (26.73)	69.87* (41.19)	67.65 (16.76)	46.39 (8.11)	45.61 (7.23)
**Multipara**	83.51 (19.70)	47.18 (45.51)	48.04 (28.78)	75.85 (18.91)	41.46 * (18.66)	60.43 * (26.11)	59.60* (45.31)	61.72 (20.75)	46.23 (8.90)	44.93 (7.43)
**Previous caesarean section**										
**No**	84.00 (18.76)	53.70 * (43.00)	48.56 (27.96)	77.93 (17.35)	44.68 (18.61)	64.26 (26.64)	65.81 (43.00)	65.73 (18.41)	46.45 (8.14)	45.28 (7.37)
**Yes**	83.75 (22.01)	40.62 * (46.59)	49.00 (29.88)	76.92 (18.79)	40.00 (18.56)	58.85 (25.91)	63.89 (18.41)	61.42 (20.59)	45.50 (10.22)	45.60 (6.97)
**Previous abortion**										
**No**	84.44 (19.46)	53.39 (43.63)	49.56 (28.88)	78.40 (17.70)	45.10 (18.50)	66.09 ** (26.99)	68.79 ** (42.53)	67.43 ** (18.80)	46.26 (8.20)	45.51 (7.38)
**Yes**	82.87 (18.61)	48.61 (43.75)	46.43 (26.49)	76.40 (17.11)	41.62 (18.87)	57.64 ** (24.70)	58.02 ** (44.04)	59.89 ** (19.90)	46.45 (7.38)	44.88 (7.17)
**Spanish**	84.05 (19.12)	52.85 (43.42)	48.68 (27.92)	77.91 (17.62)	44.34 (18.72)	64.41 * (26.47)	66.97 * (42.87)	65.85 * (18.37)	46.27 (8.46)	45.42 (7.37)
**Foreigner**	82.88 (20.55)	40.38 (45.87)	47.77 (31.93)	76.38 (16.61)	44.23 (18.09)	52.40 * (25.74)	47.43 * (44.39)	56.31 * (21.46)	47.01 (8.24)	43.99 (6.57)
**Assisted reproduction**										
**No**	85.16 ** (17.75)	52.78 (43.45)	48.25 (28.30)	77.34 (17.66)	44.19 (18.54)	63.93 (26.73)	64.86 (43.48)	65.30 (18.80)	46.55 (8.18)	45.11 (7.31)
**Yes**	68.65 ** (28.79)	41.34 (45.79)	53.23 (26.71)	83.58 (14.79)	42.31 (20.36)	58.65 (24.44)	74.36 (39.22)	63.38 (18.27)	43.35 (11.01)	48.01 (6.81)

PF: physical functioning; RP: role—physical; BP: bodily pain; GH: general health; VT: vitality; SF: social functioning; RE: role—emotional; MH: mental health; PCS: physical component summary; MCS: mental component summary. M: mean; * *p*-value < 0.05; ** *p*-value < 0.01.

## Data Availability

No additional data are available.
